# Ciliary Beat Frequency and Pattern: An Accessible Tool for the Screening of Primary Ciliary Dyskinesia

**DOI:** 10.3390/diagnostics16050704

**Published:** 2026-02-27

**Authors:** Elise Kaspi, Julie Mazenq, Adrien Pagin, Rana Mitri-Frangieh, Mohamed Boucekine, Karine Baumstarck, Thomas Radulesco, Justin Michel, Nadine Dufeu, Jean-Christophe Dubus, Patrice Roll, Diane Frankel

**Affiliations:** 1 Aix-Marseille University, APHM, INSERM, MMG, Timone Hospital, Cell Biology Department, 13005 Marseille, France; 2 APHM, Pediatric Pulmonology Department, Aix-Marseille University, INSERM, INRAE, C2VN, 13005 Marseille, France; 3 CHU Lille, Service de Toxicologie et Génopathies, 59000 Lille, France; 4 Hôpital Intercommuncal de Créteil, Department of Anatomy, Cytology and Pathology, 94000 Créteil, France; 5 Support Unit for Clinical Research and Economic Evaluation, Research Unit EA 3279, CEReSS-Health Service Research and Quality of Life Center, Department of Clinical Research and Innovation, Assistance Publique-Hôpitaux de Marseille, Aix-Marseille University, 13005 Marseille, France; 6 Aix-Marseille University, APHM, CNRS, IUSTI, La Conception University Hospital, ENT-HNS Department, 13005 Marseille, France; 7 Department of Respiratory Medicine, Aix-Marseille University, APHM, Hôpital Nord, 13015 Marseille, France

**Keywords:** primary ciliary dyskinesia, ciliary beat frequency, electron microscopy, molecular biology

## Abstract

**Background/Objectives**: Primary ciliary dyskinesia (PCD) is a rare inherited disorder caused by dysfunction of motile cilia, leading to chronic respiratory disease. Diagnosis is challenging due to heterogeneous and non-specific clinical manifestations and the absence of a single definitive diagnostic test. Current diagnostic strategies rely on a combination of functional, ultrastructural, and genetic analyses. The objective of this study was to evaluate whether ciliary beat frequency (CBF), combined with ciliary beat pattern (CBP) assessment using digital high-speed video microscopy (DHSV), could serve as an effective first-line screening tool to identify patients requiring further diagnostic investigations. **Methods**: This single-center retrospective study included 65 patients (52 children and 13 adults) with clinical suspicion of PCD. Ciliary beat analysis was performed on nasal or bronchial samples using DHSV and Sisson–Ammons Video Analysis software. CBF and CBP were assessed and compared between patients with confirmed PCD and those in whom PCD was excluded based on transmission electron microscopy (TEM) and/or molecular genetic analysis. **Results**: Fifteen patients were diagnosed with PCD. Mean CBF was significantly lower in the PCD group compared with the non-PCD group (3.3 Hz vs. 8.1 Hz; *p* < 0.001). A CBF cut-off value of 5.25 Hz yielded a sensitivity of 78.6% and a specificity of 95.7%. Three patients with PCD had CBF values above this threshold; however, two of them exhibited abnormal CBP. Sample type, patient age, and the presence of airway pathogens did not significantly influence CBF measurements. **Conclusions**: CBF and CBP analysis using DHSV represents a useful first-line screening tool within a multifaceted diagnostic approach for PCD, allowing rapid identification of patients who should undergo further confirmatory testing.

## 1. Introduction

Primary ciliary dyskinesia (PCD) is a rare genetic disease characterized by the dysfunction of motile cilia. These motile cilia are notably found in the epithelia of the upper and lower respiratory tracts (lungs and paranasal sinuses) as well as in the reproductive system and Eustachian tubes. The resulting symptoms include neonatal respiratory distress, recurrent pulmonary infections, chronic cough, and recurrent upper respiratory tract infections such as chronic otitis and rhinosinusitis. In the medium to long term, patients could develop bronchiectasis and nasal polyposis. Fertility problems and conductive hearing impairment may also be found. These symptoms, however, remain relatively non-specific, and the diagnostic delay can be quite prolonged in these patients. Some clinical features can overlap with cystic fibrosis, immunodeficiency, asthma or recurrent viral respiratory infections. The association of situs inversus with respiratory symptoms is highly indicative of PCD and is known as Kartagener’s syndrome [1,2].

Diagnosis relies on a combination of tests, including nasal nitric oxide (nNO) measurement, digital high-speed video microscopy (DHSV) to assess ciliary beat frequency (CBF) and ciliary beat pattern (CBP), transmission electron microscopy (TEM), and genetic analysis [3,4]. However, none of these tests alone has sufficient diagnostic sensitivity and specificity to serve as a definitive “gold standard” test. Environmental pollutants or infections can induce non-specific ciliary changes, leading to alterations in ciliary beat frequency and abnormalities on electron microscopy. Additionally, approximately 30% of patients do not present axonemal defects on TEM.

To date, more than 50 genes with pathogenic variants causing PCD have been identified [5]. The mode of inheritance varies depending on the gene involved and can be autosomal recessive, autosomal dominant, or X-linked. The large number of potentially implicated genes contributes to the variability of symptoms and the challenges of diagnosis. A genetic cause can be identified in approximately 70% of patients [6]. Given these complexities, multiple complementary tests are still required for an accurate diagnosis of PCD. These tests can be performed sequentially as part of a stepwise diagnostic approach [3,4,7].

In routine clinical practice at the Assistance Publique-Hôpitaux de Marseille (AP-HM, France), patients suspected of having PCD undergo a comprehensive immunological workup, a sweat test, and ciliary beat analysis to measure beat frequency and analyze beat pattern using digital high-speed video microscopy (DHSV) with the Sisson-Ammons Video Analysis (SAVA) software [8]. Nasal nitric oxide (nNO) measurement is rarely performed in our healthcare facility because of the difficulty in accessing this material.

The aim of this study was to assess whether ciliary beat frequency, in combination with ciliary beat pattern analysis using high-speed digital video microscopy, can serve as an effective screening tool to identify patients who require further diagnostic testing, including TEM and molecular analysis.

## 2. Materials and Methods

### 2.1. Patients

This study is a single-institution non-interventional investigation using retrospective data collected during the initial diagnostic work-up of patients.

We included adults and children, with a clinical and paraclinical suspicion of primary ciliary dyskinesia based on their history of neonatal respiratory distress and/or recurrent ear, nose, and throat infections, along with associated pulmonary conditions such as difficult-to-treat asthma or bronchiectasis.

For children, bronchoscopy with bronchoalveolar lavage and bronchial biopsies were performed for histopathological analysis and assessment of ciliary beating using light microscopy and ciliary ultrastructural analysis by transmission electron microscopy. Patients were generally treated with amoxicillin-clavulanate one week before the bronchoscopy. Genetic analysis was conducted if the clinical and paraclinical findings were consistent with PCD. These children were followed up at the Reference Center for Rare Respiratory Diseases in Children at the University Hospital of Timone-Enfants in Marseille (AP-HM), a center that is member of the national RespiRare network.

For adults, patients with a clinical suspicion of ciliary dyskinesia were considered eligible for nasal sampling provided that no contraindications were present (e.g., significant coagulation disorders or anticoagulant therapy). Patients were placed on a one-week course of antibiotic therapy with amoxicillin-clavulanate prior to the procedure in order to reduce upper respiratory bacterial colonization. The procedure was performed in an in-office surgical setting. Local anesthesia was achieved by applying a cotton pad saturated with xylocaine-naphazoline for 10 min. Following adequate anesthesia, a ciliary brushing was performed on both inferior nasal turbinates, targeting the posterior two-thirds of the nasal lateral wall. The brush was immediately immersed in a sterile culture medium (FertiCult IVF Medium (FertiPro nv, Beernem, Belgium)), to preserve cell viability and ciliary function [3]. Subsequently, a micro-biopsy was obtained from the same anatomical area on one of the inferior turbinates for ultrastructural examination of the cilia via transmission electron microscopy.

Patients were retrospectively included if ciliary beat frequency measurement was performed using the SAVA software (version 2.1.0W) and if electron microscopy and/or genetic analysis results were available. Clinical and biological data were collected. The diagnosis of primary ciliary dyskinesia was established by molecular biology and/or TEM results and discussed in national multidisciplinary team meeting (Multidisciplinary Consultation Meeting on Primary Ciliary Dyskinesia).

### 2.2. Ciliary Beat Frequency and Pattern

Samples (bronchial biopsy, bronchial or nasal brushing) were placed in FertiCult IVF Medium (FertiPro nv, Beernem, Belgium) immediately after collection and transported to the Cell Biology Laboratory within two hours. Each sample was placed between a microscope slide and a coverslip with sufficient Ferticult medium to ensure proper ciliary motility. Observations were performed at room temperature using a brightfield microscope with a 40× objective. (DM 2500, Leica, Nanterre, France). Videos were recorded using SAVA software with the following parameters: video length 2.1 s, number of frames 256, frame rate 120 fps, maximum measurable frequency 60 Hz, and frequency resolution 0.469 Hz. The number of recorded videos depended on the sample quality and the number of analyzable areas. Ciliary beat frequency was measured in representative regions of interest (ROIs). The average ciliary beat frequency was calculated from all the frequencies measured in each ROI.

The ciliary beat pattern was assessed by expert biologists and categorized as normal (coordinated and wave-like), dyskinetic (irregular, circular, stochastic), or immotile, based on ciliary coordination, amplitude (limited movement), and beat direction.

### 2.3. Electron Microscopy

All samples for electron microscopy were fixed in a 2.5% glutaraldehyde solution in 0.1 M cacodylate buffer. This initial fixation must last at least 4 h, and samples were stored at 4 °C. Post-fixation was then performed with osmic acid. The samples were subsequently embedded in polymerized resin. A semi-thin section was then prepared from each block with a thickness of 800 nm. Semi-thin sections were stained with 1% Azure II and 1% Methylene Blue in 1% Borax. These semi-thin sections (for optical microscopy) were examined in order to select the appropriate region for transmission electron microscopy (TEM). The region of interest was cut into an ultrathin section with a thickness of 80 nm. These sections were placed on a 200-mesh copper grid, which was then stained with saturated uranyl acetate and lead citrate. The grid was examined using a TEM microscope (HT7700, Hitachi, Tokyo, Japan). Images were interpreted with a quantitative analysis grid that analyzes each component of the axonemal structure of the cilia (central pair, central sheath, radial spokes, inner and outer dynein arms, nexin links). The overall percentage of anomalies was estimated, with a focus on the main anomalies.

### 2.4. Molecular Biology

DNA was extracted from patients’ peripheral blood leukocytes and whole exome sequencing was performed using Illumina DNA Prep Enrichment (Illumina, San Diego, CA, USA). A 150 base pairs paired-end exome sequencing was performed on an Illumina NovaSeq 6000 platform. Reads were aligned to the Genome Reference Consortium human genome build 38 (GRCh38) and analyzed against a restricted in silico panel of 48 to 55 genes involved in bronchiectasis (depending on the time of analysis) using the in-house ANATOLE pipeline. The latest version of the panel includes 49 genes involved in PCD (*CCDC39*, *CCDC40*, *CCDC65*, *CCDC103*, *CCNO*, *CFAP74*, *CFAP221*, *CFAP298*, *CFAP300*, *DAW1*, *DNAAF1*, *DNAAF2*, *DNAAF3*, *DNAAF4*, *DNAAF5*, *DNAAF6*, *DNAAF11*, *DNAH5*, *DNAH9*, *DNAH10*, *DNAH11*, *DNAI1*, *DNAI2*, *DNAJB13*, *DNAL1*, *DRC1*, *FOXJ1*, *GAS2L2*, *GAS8*, *HYDIN*, *LRRC56*, *MCIDAS*, *NEK10*, *NME5*, *NME8*, *ODAD1*, *ODOD2*, *ODAD3*, *ODAD4*, *OFD1*, *RSPH1*, *RSPH3*, *RSPH4A*, *RSPH9*, *SPAG1*, *STK36*, *TP73*, *TTC12* and *ZMYND10*) and six genes involved in cystic fibrosis, atypical forms or differential diagnosis (*CA12*, *CFTR*, *SCNN1A*, *SCNN1B*, *SCNN1G* and *SLC26A9*). Variants were prioritised according to their frequency in the general population (GnomAD database and local data), published cases (HGMDpro, PubMed), bioinformatic predictions (Mobidetails, SpliceAI-visual, etc.) and classified according to the American College of Medical Genetics (ACMG) guidelines [9,10,11].

### 2.5. Statistical Analysis

Descriptive analyses were performed to summarize the variables. Continuous variables were presented as mean and standard deviation (SD), whereas categorical variables were expressed as absolute frequencies and percentages. Comparisons of continuous variables between groups were conducted using the Mann–Whitney U test for two-group comparisons and the Kruskal–Wallis test for comparisons involving more than two groups. Categorical variables were compared using the Chi-square test or Fisher’s exact test, as appropriate.

A Receiver Operating Characteristic (ROC) curve analysis was performed to determine the optimal cutoff value of ciliary beat frequency mean for discriminating between the two groups (non-PCD group and PCD group). The Youden index method was applied to identify the threshold that maximized both sensitivity and specificity. The AUC, sensitivity, specificity, positive predictive value (PPV), and negative predictive value (NPV) were reported.

To explore factors associated with Ciliary beat frequency mean and its standard deviation, univariate and multivariate linear regression analyses were conducted. The results were expressed as beta coefficients (β), 95% confidence intervals (95% CI), and *p*-values. A *p*-value < 0.05 was considered statistically significant. All statistical analyses were performed using R software (version 4.4.1).

AI-assisted language editing tools were used during the preparation of this manuscript to improve the English language and grammar.

## 3. Results

This study included 65 patients, 52 children (mean age 6 years old, range 0–16) and 13 adults (mean age 41 years old, range 24–67), 34 female (52%) and 31 male (48%).

### 3.1. Identification of PCD Patients by TEM and Molecular Analysis

**Figure 1:**
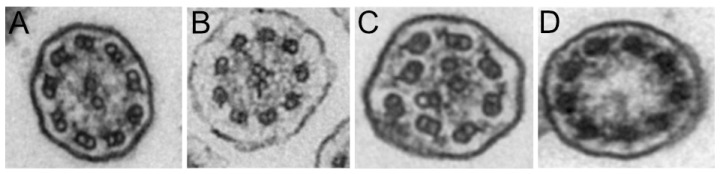
Representative transmission electron microscopy images of (**A**) outer dynein arm (ODA) defects, (**B**) outer and inner dynein arm defects, (**C**) disorganization of the central pair complex, and (**D**) absence of the central pair complex.

TEM was performed on 52 patients (80%). The results were classified as normal or borderline normal for 36 patients (55%) and inconclusive for 7 patients (13%). In the latter case, this was mainly due to ciliary abrasion, which hindered the analysis of the ciliary ultrastructure. For 9 patients (17%), ultrastructural anomalies were identified, including the absence of the outer dynein arm, with or without absence of the inner dynein arm (5 patients, Figure 1A,B). Disorganization or absence of the central pair complex was found in 4 patients (Figure 1C,D).

Genetic testing was performed in 41 cases (63%), and mutations were detected in 14/41 patients (34%). Among these 14 patients, ten were diagnosed with PCD (Table 1). Two patients had biallelic pathogenic variants in the dynein axonemal heavy chain 5 (*DNAH5*). Other mutations identified are listed in Table 1 and include dynein axonemal heavy chain 11 (*DNAH11*), dynein axonemal intermediate chain 1 (*DNAI1*), cilia and flagella-associated protein 300 (*CFAP300*), radial spoke head component 1 (*RSPH1*), cyclin O (*CCNO*), dynein axonemal intermediate chain 2 (*DNAI2*), coiled-coil domain 39 molecular ruler complex subunit (*CCDC39*), and DnaJ heat shock protein family (*Hsp40*) member B13 (*DNAJB13*). For one patient, PCD diagnosis remains uncertain due to the presence of an homozygous variant of uncertain significance on the NIMA-related kinase 10 (*NEK10*) gene and normal ciliary structure and mobility, as previously reported for *NEK10* patients [12,13,14].

For two patients, only one heterozygous mutation associated with the pathophysiology of PCD was identified: a nonsense mutation in the dynein axonemal heavy chain 9 (*DNAH9* c.11299C>T, p.Arg3767*) classified as pathogenic in one patient, and a missense mutation in the outer dynein arm docking complex subunit 1 (*ODAD1* c.742G>A, p.Ala248Serfs*52) also classified as pathogenic in the other. As no second mutation was identified, these two patients were not considered to have PCD. In another patient, heterozygous mutations in Cilia and Flagella Associated Protein 54 (*CFAP54*) c.7346A>G, p.His2449Arg and c.8771T>G, p.Thr2924Met were identified, but these mutations were not classified as pathogenic for PCD.

Based on the results of TEM and genetic testing, 15 patients were diagnosed with PCD (Table 1).

### 3.2. Results of Ciliary Beat Analysis with Digital High-Speed Video Microscopy

We analyzed the ciliary beat frequency in 65 patients at room temperature using the SAVA system and categorized them into two groups based on genetic testing and TEM. The first group included 15 patients with PCD (PCD group), while the second group included 50 patients in whom PCD was excluded (non-PCD group).

**Figure 2:**
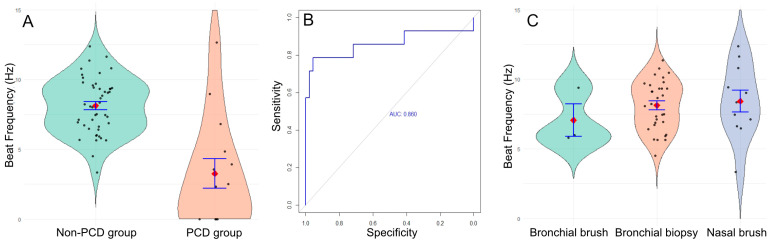
(**A**) Mean ciliary beat frequency in the non-PCD group (green) and the PCD group (orange). (**B**) ROC curve. (**C**) Ciliary beat frequency in bronchial brushes (green, *n* = 3), bronchial biopsies (orange, *n* = 35), and nasal brushes (blue, *n* = 12).

We found a significant decrease in ciliary beat frequency in the PCD group, with a mean of 3.3 Hz (IQR 0–4.6, range 0–12.7), compared to the non-PCD group, which had a mean of 8.1 Hz (IQR 6.7–9.4, range 3.3–12.4) (*p* < 0.001; Mann–Whitney U test, Figure 2A).

Using these results, we calculated the sensitivity and specificity of the technique and found a sensitivity of 78.6% and a specificity of 95.7%, with a positive predictive value of 93.6%, a negative predictive value of 84.6%, and an area under the curve of 0.86. The optimal threshold to distinguish PCD from non-PCD was estimated at 5.25 Hz (Figure 2B). Using this cut-off, 3 patients diagnosed with PCD would have been misclassified (CBF values of 12.7 ± 4.5; 9.0 ± 0.9 and 6.8 ± 3.8 Hz). However, for two of them, the ciliary beat pattern showed heterogeneous beating, which should have prompted further diagnostic testing for PCD (videos in Supplemental Data). The third one was the patient with the homozygous mutation in *NEK10* (videos in Supplemental Data).

We sought to determine whether the sample type influenced ciliary beat frequency results. To investigate this, we compared the mean ciliary beat frequency across different sample types in the non-PCD group: nasal brush (*n* = 12; 8.42 Hz), bronchial brush (*n* = 3; 7.07 Hz), and bronchial biopsy (*n* = 35; 7.65 Hz). No significant difference was found (*p* = 0.530, Fisher’s exact test, Figure 2C), indicating that these sample types are comparable for the study of ciliary beating using DHSV. However, the standard deviation was higher for nasal brush (2.62 Hz) compared to bronchial biopsy (1.34 Hz) and bronchial brush (1.29 Hz) (*p* = 0.025, Fisher’s exact test), suggesting that bronchial samples are more homogeneous than nasal samples. Moreover, ciliary abrasion was twice as common in nasal brushings (5/19, 26.3%) compared to bronchial biopsies (5/40, 12.5%).

### 3.3. Other Clinical and Biological Results

Situs inversus and consanguinity were observed exclusively in the PCD group (7 patients (50%) and 3 patients (25%), respectively; see Supplemental Table S1). Neonatal respiratory distress was reported in 6 patients, 3 in the PCD group and 3 in the non-PCD group. Otolaryngologic and pulmonary symptoms were more frequently observed in the PCD group (*p* = 0.06 and *p* = 0.043, respectively, Fisher’s exact test), whereas bronchiectasis was not significantly different between groups (*p* = 0.904, Fisher’s exact test). Asthma was more prevalent in the non-PCD group (*p* = 0.001, Fisher’s exact test). Additionally, we verified that age had no significant effect on ciliary beat frequency (beta = 0.02, *p* = 0.5, multivariate linear regression).

Bronchoalveolar lavage (BAL) was performed at the same time as bronchial biopsy in 43 patients, including 38 patients without PCD and 5 patients with PCD. In all 5 PCD cases, BAL revealed the presence of pathogens and an inflammatory response characterized by neutrophils predominance on cytological analysis, despite receiving antibiotic treatment in the week prior to ciliary examination (see Supplemental Table S1). We confirmed that the presence of pathogens at the time of analysis did not influence ciliary beat frequency (beta = 1.3, *p* = 0.056, multivariate linear regression).

## 4. Discussion

Our study aimed to determine whether the quantitative and qualitative analysis of ciliary beating could be an effective screening tool for the diagnosis of primary ciliary dyskinesia. Diagnosis of primary ciliary dyskinesia is challenging due to wide clinical variability and overlapping features with other respiratory conditions.

In this study, we analyzed data from 65 patients in whom the diagnosis of PCD was either confirmed or excluded using transmission electron microscopy and/or molecular biology. Our findings indicate that CBF analysis demonstrates a sensitivity of 78.6% and a specificity of 95.7%, making it a valuable tool for initial screening, rather than a definitive diagnostic test. The optimal cut-off value to discriminate between PCD and non-PCD patients was determined to be 5.25 Hz. Interestingly, three patients diagnosed with PCD had values above this threshold. However, two of them exhibited abnormal ciliary beating patterns, which should have prompted further investigation.

Ciliary beat frequency analysis is a relatively simple test to perform and provides a rapid turnaround time for results, as they can be obtained on the same day as the sample collection. This allows clinicians to make quick decisions about whether to proceed with further diagnostic testing, such as TEM or genetic analysis. However, the lack of consensus on standard operating procedures for sample collection and measurement of ciliary beat frequency limits the validation of this technique as a diagnostic procedure [3,7,15]. Standardization is essential to establish reference ranges. For instance, we performed our CBF measurements at room temperature because we did not have a heated stage to maintain the recommended temperature of 37 °C [16,17]. This resulted in a mean CBF of 8.8 Hz in the non-PCD group, which is consistent with other studies [18]. In comparison, Lee et al. reported a mean CBF of 10.1 Hz at 37 °C in 135 healthy children using nasal brushing. Another study found a mean CBF of 12.8 Hz in pediatric patients and 11.5 Hz in adults, also at 37 °C [19]. The variation in frequency values indicates the necessity for establishing standard values for the specific conditions under which measurements are conducted in each laboratory. Despite these variations, CBF analysis using DHSV remains a sensitive technique for detecting abnormalities in ciliary function. Rubbo et al. analyzed videos of 120 patients with DHSV and found a sensitivity of 100% and a specificity of 96%, respectively for the diagnosis of PCD compared with ERS guidelines [20]. While ciliary motility is known to be temperature-dependent, the screening of CBP at room temperature remains effective for detecting major functional abnormalities, such as immotile or severely dyskinetic cilia. Analysis of CBP, including coordination, amplitude, and beat direction, allows reliable identification of these defects even when subtle changes may be influenced by lower temperatures. Nevertheless, we acknowledge that some temperature-sensitive features, such as metachronal wave propagation or minor variations in beat amplitude, may be attenuated at room temperature, and interpretation of subtle or borderline findings should consider this limitation.

Nasal brushing is the most accessible procedure for obtaining ciliary cells; however, the nasal cavities are more exposed to chronic inflammation and pollutants than the bronchi [21]. In our study, we obtained more uninterpretable results due to the absence of cilia in nasal brushings (26.3%) than in bronchial biopsies (12.5%). Moreover, the standard deviation was higher with nasal brushings (2.62 Hz) compared to bronchial samples (1.34 Hz). For one patient diagnosed with PCD, the CBF measurement could not have been performed due to the absence of cilia on the epithelial cells. These findings were confirmed by TEM. Nevertheless, we did not find any difference in CBF between the sample types used in our study (nasal or bronchial brushing, bronchial biopsy), which is consistent with other studies [22,23]. Moreover, nasal brushing is the simplest technique for both adults and children and can be easily repeated if the result is inconclusive.

Molecular testing revealed a *CCNO* mutation which is known to lead to a reduced generation of multiple motile cilia, explaining the results found by light microscopy and TEM [24]. We identified a homozygous *NEK10* mutation (c.2095G>A, p.Glu699Lys) of unknown significance in one patient. This gene has been implicated in PCD by several studies, which reported normal CBF and TEM results [12,13,14].

This study has several limitations. First, due to access constraints, we were unable to measure nasal nitric oxide (nNO) levels in patients. Additionally, with regard to ciliary beat analysis, our laboratory lacks the equipment required to assess ciliary beat frequency at 37 °C, as recommended by international guidelines. Therefore, this study is not intended to establish a definitive diagnosis based solely on ciliary beat frequency and pattern analysis. Rather, as part of a multifaceted diagnostic approach, ciliary beat frequency analysis can be considered a useful first-line investigative step [17].

## 5. Conclusions

Our study demonstrates the value of using DHSV and CBF measurement for PCD initial screening. This test is effective in identifying patients who require further confirmatory testing, such as TEM or molecular analysis. Additionally, we showed that the type of sample, patient age, and the presence of pathogens had no statistically significant effect on CBF. This cost-effective technique provides a preliminary result on the same day as sample collection and can be easily implemented in a laboratory setting. This technique is not intended to replace TEM or genetic testing. In any case, when ciliary beat analysis suggests PCD, or when CBF is normal but clinical symptoms are suggestive, further testing must be performed.

## Figures and Tables

**Table 1:** Clinical and biological data of PCD patients. Id.: patient identification; SI: situs inversus; Consang.: consanguinity; CBF: ciliary beat frequency; CBP: ciliary beat pattern; TEM: transmission electron microscopy; ODA: outer dynein arm; IDA: inner dynein arm. * indicates a premature stop codon generated by the frameshift mutation.

Id.	SI	Consang.	Neonatal Respiratory Distress	Bronchiectasis	ENT Symptoms	Pulmonary Symptoms	Asthma	Type of Sample	Mean CBF (Hz)	CBP/CBF	TEM	Genetic Testing	
												Allele 1	Allele 2
1	Yes	No	No	No	Yes	Yes	No	Bronchial biopsy	0.0 ± 0.0	Static	Combined IDA and ODA absence from 25–50% of cross sections	not performed	
2	Yes	No	No	No	Yes	Yes	No	Nasal brush	12.7 ± 4.5	Highly heterogeneous frequency and pattern	5% variable anomalies	*DNAH11* c.13306G>A, p.Ala4436Thr (likely pathogenic)	*DNAH11* c.13373C>T, p.Pro4458leu (pathogenic)
3	Yes	No	Yes	No	Yes	No	Yes	Nasal brush	0.0 ± 0.0	Static		*DNAH5* c.4636C>T, p.Gln1546Ter (pathogenic)	*DNAH5* c.5114+1G>C, p.? (pathogenic)
4	Yes	No	Yes	No	Yes	No	No	Nasal brush	2.3 ± 0.1	Mostly static	4% minor anomalies	*DNAH11* c.8698C>T, p.Arg2900Ter (pathogenic)	*DNAH11* c.8698C>T, p.Arg2900Ter (pathogenic)
5	Yes	No	No	Yes	Yes	Yes	Yes	Bronchial biopsy	0.0 ± 0.0	Static	100% ODA absence	*DNAI1* c.l0l9+5G>C, p.? (pathogenic)	*DNAI1* c.l0l9+5G>C, p.? (pathogenic)
6	Yes	No	No	No	Yes	No	No	Nasal brush	0.0 ± 0.0	Mostly static	ODA absence	not performed	
7	No	Yes	No	Yes	Yes	Yes	No	Bronchial biopsy	0.0 ± 0.0	Static	ODA and IDA absence	*CFAP300* c.(608+1_ 609-1) _804+ ?del, p.? (likely pathogenic)	*CFAP300* c.(608+1_ 609-1) _804+ ?del, p.? (likely pathogenic)
8	No	No	Yes	Yes	Yes	Yes	No	Bronchial biopsy	4.9 ± 1.4	Dyskinetic	Central complex defect	*RSPH1* c.275-2A>C, p.? (pathogenic)	*RSPH1* c.281G>A, p.Trp94Ter (pathogenic)
9				Yes	Yes	Yes	No	Nasal brush	/	Absence of cilia	Epithelium abrasion	*CCNO* c.258_262dup, p.Gln88Argfs*8 (pathogenic)	*CCNO* c.258_262dup, p.Gln88Argfs*8 (pathogenic)
10	Yes	No	No	No	Yes	No	Yes	Nasal brush	0.0 ± 0.0	Mostly static		*DNAI2* c.740G>A, p.Ag247Gln (pathogenic)	*DNAI2* c.1516C>T, p.Arg506Ter (pathogenic)
11	No			Yes	Yes	Yes	No	Nasal brush	3.9 ± 2.2	Mostly static or with low frequency	Microtubular disorganisation and IDA absence	*CCDC39* c.1167+1261A>G, p.? (pathogenic)	*CCDC39* c.1768C>T, p.Gln590Ter (pathogenic)
12	No	Yes		Yes	Yes	Yes	No	Nasal brush	6.8 ± 3.8	Low number of cilia. Beats are rather heterogeneous or even absent	Central complex defect (absence)	*DNAJB13* c.68+1G>C, p.? (pathogenic)	*DNAJB13* c.68+1G>C, p.? (pathogenic)
13	No			Yes	Yes	Yes	No	Nasal brush	2.5 ± 0.9	Low frequency	ODA absence ± Central complex defect	*DNAH5* c.11930del, p.Asn3977Thrfs Ter54 (pathogenic)	*DNAH5* c.11930del, p.Asn3977ThrfsTer54 (pathogenic)
14	No	No	No	No	Yes	No	No	Nasal brush	3.5 ± 0.6	Low frequency	ODA defect	not performed	
15	No	Yes	No	Yes	Yes	Yes	No	Bronchial biopsy	9.0 ± 0.9	Normal	Normal	*NEK10* c.2095G>A p.Glu699Lys (unknown significance)	*NEK10* c.2095G>A p.Glu699Lys (unknown significance)

## Data Availability

All data generated or analyzed during this study are included in this article and its Supplementary Material Files. Further enquiries can be directed to the corresponding author.

## Supplementary Materials

The following supporting information can be downloaded at: https://www.mdpi.com/article/10.3390/diagnostics16050704/s1, Video S1: Normal, Video S2: case 2, Video S3: case 10, Video S4: case 15; Supplemental Table S1: clinical and biological data. F: female; M: Male; BAL: bronchoalveolar lavage; TEM: transmission electron microscopy.

## Abbreviations

The following abbreviations are used in this manuscript:

PCD	primary ciliary dyskinesia
CBF	Ciliary beat frequency
CBP	Ciliary beat pattern
DHSV	digital high-speed video microscopy
TEM	Transmission electron microscopy
