# Supplementary material for: Ciliary Beat Frequency and Pattern: An Accessible Tool for the Screening of Primary Ciliary Dyskinesia
Source: Diagnostics (Basel). 2026 Feb 27;16(5):704. doi: 10.3390/diagnostics16050704 (PMC12984594; doi:10.3390/diagnostics16050704)
Supplement: Supplementary file 1 [file diagnostics-16-00704-s001.zip › Supplemental Table S1.pdf]

Characteristic	Total: n=65[1]	Non-PCD group (n=50)	PCD group (n=15)	p-value[2]
**Age**				0.314
Median (IQR)	6 (3 - 15)	6 (3 - 13)	7 (5 - 34)	
Mean (SD)	13 (15)	12 (15)	18 (18)	
Range	0 - 67	0 - 67	0 - 48	
**Sexe**				0.093
F	34 (52%)	29 (58%)	5 (33%)	
M	31 (48%)	21 (42%)	10 (67%)	
Situs inversus				**<0.001**
Absence	55 (89%)	48 (100%)	7 (50%)	
Presence	7 (11%)	0 (0%)	7 (50%)	
Unknown	3	2	1	
**Consanguinity**				**0.006**
Absence	57 (95%)	48 (100%)	9 (75%)	
Presence	3 (5.0%)	0 (0%)	3 (25%)	
Unknown	5	2	3	
**Neonatal respiratory distress**				0.072
Absence	53 (90%)	45 (94%)	8 (73%)	
Presence	6 (10%)	3 (6.3%)	3 (27%)	
Unknown	6	2	4	
**Bronchiestasy**				0.904
Absence	29 (45%)	22 (45%)	7 (47%)	
Presence	35 (55%)	27 (55%)	8 (53%)	
Unknown	1	1	0	
**Otolaryngologic symptoms**				**0.006**
Absence	18 (28%)	18 (36%)	0 (0%)	
Presence	47 (72%)	32 (64%)	15 (100%)	
**Pulmonary symptoms**				**0.043**
Absence	10 (15%)	5 (10%)	5 (33%)	
Presence	55 (85%)	45 (90%)	10 (67%)	
**Smoke**				0.619
Non smoker	43 (73%)	31 (69%)	12 (86%)	
Smoker	1 (1.7%)	1 (2.2%)	0 (0%)	
Passive	11 (19%)	10 (22%)	1 (7.1%)	
Smoke-free	4 (6.8%)	3 (6.7%)	1 (7.1%)	
Unknown	6	5	1	
**Asthma**				**0.001**
Absence	28 (44%)	16 (33%)	12 (80%)	
presence	36 (56%)	33 (67%)	3 (20%)	
Unknown	1	1	0	

**Characteristic**	Total: n=65[1]	Non-PCD group (n=50)	PCD group (n=15)	**p-value**[2]
**Sweat test**				
negative	45 (100%)	38 (100%)	7 (100%)	
Unknown	20	12	8	
***CFTR* mutation**				0.653
Negative	31 (79%)	22 (76%)	9 (90%)	
Heterozygous	8 (21%)	7 (24%)	1 (10%)	
Unknown	26	21	5	
**Germs in BAL**				0.153
Absence	15 (33%)	15 (38%)	0 (0%)	
Presence	30 (67%)	25 (63%)	5 (100%)	
Unknown	20	10	10	
**Cytological analysis of BAL**				0.220
Normal	17 (40%)	17 (45%)	0 (0%)	
Inflammatory (with neutrophils)	22 (51%)	17 (45%)	5 (100%)	
Inflammatory (with lymphocytes)	2 (4.7%)	2 (5.3%)	0 (0%)	
Non conclusive	1 (2.3%)	1 (2.6%)	0 (0%)	
hemorrhagic	1 (2.3%)	1 (2.6%)	0 (0%)	
**TEM**				**<0.001**
Normal	28 (54%)	25 (64%)	3 (23%)	
borderline normal	8 (15%)	8 (21%)	0 (0%)	
abnormal	9 (17%)	0 (0%)	9 (69%)	
Inconclusive	7 (13%)	6 (15%)	1 (7.7%)	
Not performed	13	11	2	
**Genetic testing**				**<0.001**
Not conclusive	27 (66%)	27 (90%)	0 (0%)	
Conclusive	14 (34%)	3 (10%)	11 (100%)	
Not performed	24	20	4	
**Beat frequency (Hz)**				**<0.001**
Median (IQR)	7.4 (5.7 - 9.3)	8.2 (6.7 - 9.4)	2.4 (0.0 - 4.6)	
Mean (SD)	7.0 (3.3)	8.1 (2.0)	3.3 (3.9)	
Range	0.0 - 12.7	3.3 - 12.4	0.0 - 12.7	
Non-evaluable	5	4	1	
**Beat frequency conclusion**				**<0.001**
Normal	42 (76%)	41 (100%)	1 (7.1%)	
Abnormal	13 (24%)	0 (0%)	13 (93%)	
Inconclusive	10	9	1	

[1]n (%)

[2]Pearson's Chi-squared test; Fisher's exact test; Wilcoxon rank sum test

Supplemental Table S1: clinical and biological data. F: female; M: Male; BAL: bronchoalveolar lavage; TEM: transmission electron microscopy.
